# Gigantism and Its Implications for the History of Life

**DOI:** 10.1371/journal.pone.0146092

**Published:** 2016-01-15

**Authors:** Geerat J. Vermeij

**Affiliations:** Department of Earth and Planetary Sciences, University of California, One Shields Avenue, Davis, California, 95616, United States of America; University of Naples, ITALY

## Abstract

Gigantism—very large body size—is an ecologically important trait associated with competitive superiority. Although it has been studied in particular cases, the general conditions for the evolution and maintenance of gigantism remain obscure. I compiled sizes and dates for the largest species in 3 terrestrial and 7 marine trophic and habitat categories of animals from throughout the Phanerozoic. The largest species (global giants) in all categories are of post-Paleozoic age. Gigantism at this level appeared tens to hundreds of millions of years after mass extinctions and long after the origins of clades in which it evolved. Marine gigantism correlates with high planktic or seafloor productivity, but on land the correspondence between productivity and gigantism is weak at best. All global giants are aerobically active animals, not gentle giants with low metabolic demands. Oxygen concentration in the atmosphere correlates with gigantism in the Paleozoic but not thereafter, likely because of the elaboration of efficient gas-exchange systems in clades containing giants. Although temperature and habitat size are important in the evolution of very large size in some cases, the most important (and rare) enabling circumstance is a highly developed ecological infrastructure in which essential resources are abundant and effectively recycled and reused, permitting activity levels to increase and setting the stage for gigantic animals to evolve. Gigantism as a hallmark of competitive superiority appears to have lost its luster on land after the Mesozoic in favor of alternative means of achieving dominance, especially including social organization and coordinated food-gathering.

## Introduction: Where, When, and How to Be a Giant

There is something alluring and faintly threatening about giants. Dinosaurs and other gigantic fossils have fired the popular imagination, and there is no shortage of speculation about how these animals lived. Nevertheless, scientific inquiry has focused almost entirely on the particulars of individual cases of gigantism rather than on the phenomenon of very large size in general. This lack of study of gigantism throughout life's realm has to do with the nagging possibility that very large organisms owe their enormous size to unique intrinsic traits or that they represent mere statistical outliers of a size distribution that ranges over 14 orders of magnitude. In this latter view, giants reflect little more than random variation requiring no further explanation. However, it has long been known that very large plants and animals are functionally unlike their smaller counterparts: They are more likely to be top consumers or producers, to tolerate a greater range of environmental conditions (at least in the case of animals), to maintain internal homeostasis more effectively, to be less vulnerable as adults to lethal predation, to compete more successfully for mates (again mainly in animals) and to be more prone to extinction during times of crisis [1–3]. Gigantism is thus a functionally distinct and ecologically important condition that is both enabled by resources and compelled by natural selection. The distribution of maximum size in time and space can therefore inform our understanding of major patterns in the history of life.

In order to explain gigantism, it is necessary first to document its distribution, and then to consider possible factors that either make gigantism possible or that propel lineages to very large body sizes. By identifying and characterizing giants from different times, places and clades, we can ask which circumstances are conducive to the evolution and maintenance of exceptionally large organisms.

My emphasis on one extreme end of the size distribution of organisms is a deliberate attempt to move away from considerations of the whole size distribution [4–7] or of mean or optimal size in a clade [6]. Combining organisms of all sizes into analyses of spatial patterns or historical trends introduces unacknowledged functional heterogeneity, introducing artifacts and complicating interpretations of results [3]. Though easily quantified, mean size in a clade or an assemblage is about as meaningful as, say, mean color or modal shape. The idea that there is an optimal size of species is untestable and unsupported by evidence: organisms large and small exist and propagate because they work adequately in the ecosystems they occupy. Moreover, selection is not uniform for all species, and certainly not for very large and very small species. By concentrating on giants, I can focus on the conditions that favor one ecologically important trait.

Here I survey giants—the largest-bodied species in their ecological category or clade—throughout the Phanerozoic eon in ten terrestrial and marine trophic and habitat types, and examine the enabling and selective factors that collectively can account for observed patterns in very large body size. I argue that selection in favor of extreme gigantism is due largely to competitive interactions and to a lesser extent to predation, and that evolving ecological interdependencies between primary producers and consumers created the conditions that enabled some lineages of competitively superior animals to achieve exceptionally large size after the Paleozoic era. I also consider the vexing question why Mesozoic terrestrial vertebrates attained greater maximum sizes than their later Cenozoic counterparts.

## Methods

### Definitions and Analysis

Data on the sizes, spatial and temporal distribution, metabolic properties, trophic roles and phylogenetic position of extremely large organisms were gathered from the literature. I considered the following trophic categories of animals: terrestrial and marine apex predators (consumers of other large animals), terrestrial and marine herbivores (animals that consume attached macroscopic photosynthesizers, mainly plants and seaweeds), solitary marine photosymbiotic animals, marine chemosymbiotic animals, plankton-feeding non-symbiotic animals on the seafloor, and plankton-feeding pelagic (mid-water) animals. In addition, I consider patterns of gigantism over time in several major clades including molluscan classes, several groups of arthropods, and several vertebrate clades. Data for seaweeds and plants are insufficient, but gigantism in photosynthesizing organisms is also briefly discussed. I did not consider fungi or colonial marine animals.

By necessity, one species must be the largest member of its clade, trophic level or ecosystem either at a particular time or for all time. I define a local giant as the largest-bodied species in a particular place or interval of time. Species that are the largest members of their ecological (trophic and habitat) category at the global spatial scale and/or throughout the Phanerozoic eon are called global giants.

In the literature, maximum size is estimated either in linear units or as body mass. I have taken these estimates directly from published sources without further converting them to a common standard with the full realization that inferences of size often involve extrapolation from particular body parts, and that differences in shape make direct comparisons difficult. Ideally, size estimates should be based on the mass of metabolizing tissues, but that standard is rarely achieved even for living species.

In the analyses to follow, I purposely avoid a statistical treatment. The reason is simple: statistics describe the properties of and differences between distributions of populations with respect to one or more variables such as body size; whereas this paper is explicitly about the extreme end of a distribution, namely, the maximum body size within ecological, temporal or phylogenetic categories. It is therefore meaningless to ascertain whether there are statistically significant differences among categories given that each category is represented by a single point (global Phanerozoic-level giant) or up to three points (era-level giants). For similar reasons, I have eschewed discussions of Cope's Rule, the purported (and sometimes demonstrated) trend within clades and among replacing clades toward larger body size. Cope's Rule is usually tested by examining the entire size distribution within a clade over time. I would argue that the evolutionary trajectory of size within lineages is independent of that in other lineages, and that the obvious size increase leading to the largest member of a category has nothing to do with trends in other lineages or in the size distribution of the clade as a whole. Finally, I have resisted providing quantitative correlations between era-level or Phanerozoic-level gigantism and the various enabling and selective factors thought to favor very large size. Not only are precise estimates of the various factors and of the trends in those factors not well constrained, but a correlation summarized by a single number imposes a false sense of precision and obscures important caveats and variations whose recognition informs and qualifies interpretations.

For both living and fossil giants there is considerable uncertainty in the measurement of size [8, 9]. Moreover, maximum size varies within and among sites and between males and females, and is subject to the vagaries of sampling. To simplify matters, I report the largest measurements for the largest species in its category under the rationale that an individual of this very large size was able to grow and survive in at least some conditions in which the species existed.

### Establishing the Identity of Giants

Different body proportions between very large animals introduce some ambiguity into the determination of the largest species in a given category. For example, the putatively photosymbiotic Late Cretaceous rudist bivalve *Titanosarcolites* reaches a larger linear dimension (1 m) than the living *Tridacna gigas* (137 cm), but its body was likely much smaller for a given shell volume. I have therefore opted for *T*. *gigas* as the largest known solitary photosymbiotic animal. The largest Paleozoic bottom-dwelling sedentary suspension-feeders (the brachiopod *Gigantoproductus* and the hyolith *Macrotheca*) have greater linear dimensions than the largest (200 mm) Permian bivalve [9]. I have accepted the former two taxa as the largest in their category even though brachiopod tissues occupy a small fraction of the internal shell volume and the hyolith is a slender tubular animal.

The largest cephalopod in linear dimension is an unnamed Middle Ordovician straight-shelled endoceratid estimated to have been 8 to 9 m long [10, 11]; but the Late Cretaceous ammonoid *Parapuzosia seppenradensis*, with a coiled instead of a straight shell, had a diameter estimated between 2.55 and 3.5 m, which translates to a length of ~19 m if the shell were uncoiled [12, 10], comparable to the length of the living shell-less giant squid *Architeuthis dux* (19 m). The most massive living cephalopod known is the colossal squid *Mesonychoteuthis hamiltoni*, whose mass (500 kg) considerably exceeds the 200 kg of *A*. *dux* [13]. It is likely that one of the two living species mentioned should be considered the largest species, because the body chamber in which most of the animal's mass is concentrated in shell-bearing cephalopods is much shorter than the shell as a whole. Paleozoic giant cephalopods are without doubt the largest shell-bearing animals that have ever lived, but their tissue mass is likely to have been smaller than that of the largest living cephalopods, which lack an external shell.

Even for insects there is some ambiguity. The largest living beetle *Titanus giganteus* (length 17 cm) is smaller in linear dimension than the largest Carboniferous dragonfly (wingspan 71 cm) [14], but it may well exceed the fossil species in body mass. In this case I accept the fossil *Meganeuropsis permiana* as the largest insect.

## Results and Discussion

### Gigantism Over Time

**Fig 1:**
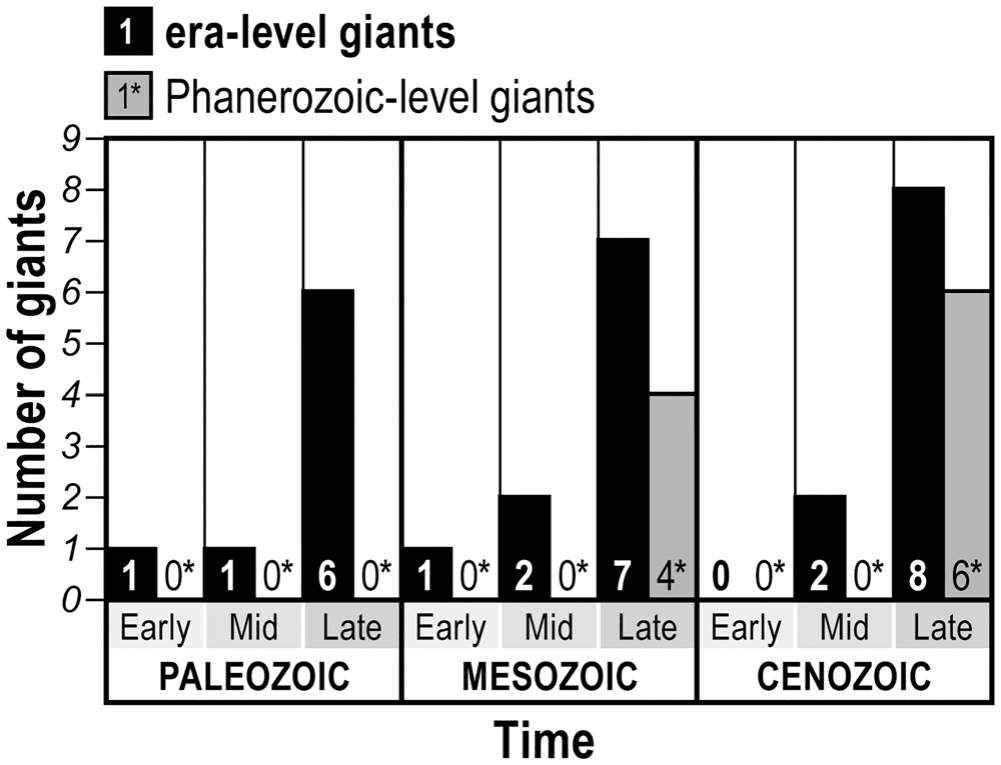
Number of era-level and Phanerozoic-level giants over time.

Table 1 and Fig 1 show that the largest animals of the Phanerozoic in all ten ecological (trophic and habitat) categories are of Mesozoic or Cenozoic age. All Phanerozoic-level global terrestrial giants occur from the mid-Cretaceous to the Late Cretaceous, but the entire 100-million-year span from the Late Jurassic to the end of the Cretaceous is marked by extreme gigantism in dinosaurs (sauropods, ornithischians, theropods, ceratopsians and hadrosaurs), crocodylians and turtles [5, 15–18].

**Table 1:** Sizes of global giants in ecological categories over time.

Category			Clade	Age	Size
Ground-dwelling terrestrial predators	Palaoezoic	*Anteosaurus*	Therapsida	Late Permian	2000 kg^[^^139^^]^
	Mesozoic	*Tyrannosaurus rex*	Archosauria	Maastrichtian	7700 kg^[^^17^^]^
	Cenozoic	*Arctotherium angustidens*	Mammalia	Early Pleistocene	983–2042 kg^[^^141^^]^
Terrestrial herbivores	Palaeozoic	*Tapinocephalus* sp.	Therapsida	Late Permian	1600–2000 kg^[^^142^^]^
	Mesozoic	*Argentinosaurus huinculensis*	Archosauria	Albian-Cenomanian	40m, 90,000 kg^[^^17^^,^ ^140^^]^
	Cenozoic	*Indricotherium transouralicum*	Mammalia	Late Oligocene	7.4m, 15–20,000 kg^[^^143^^]^
Flying predators	Palaeozoic	*Meganeuropsis permiana*	Insecta	Late Carboniferous	71 cm wingspan^[^^144^^]^
	Mesozoic	*Quetzalcoatlus northropi*	Pterosauria	Maastrichtian	10-11m wingspan^[^^145^^]^
	Cenozoic	*Pelagornis sandersi*	Aves	Late Oligocene	6.4 m wingspan^[^^146^^]^
Marine pelagic predators	Palaeozoic	*Helicoprion* sp.	Chondrichthyes	Middle Permian	10 m^[^^147^^]^
	Mesozoic	*Shonisaurus sikanniensis*	Ichthyopterygia	Norian	17–20 m^[^^148^^]^
	Cenozoic	*Physeter macrocephalus*	Mammalia	Recent	24 m, 16,500 kg^[^^8^^]^
Marine herbivores	Mesozoic	*Leviathania* sp.	Gastropoda	Late Tithonian or early Berriasian	400 mm^[^^149^^]^
	Cenozoic	*Hydrodamalis gigas*	Mammalia	Holocene	10 m, 10,000 kg^[^^138^^,^ ^63^^]^
Bottom-dwelling marine predators	Palaeozoic	Endoceratid cephalopod	Cephalopoda	Middle Ordovician	8–9 m^[^^10^^]^
	Mesozoic	*Ptychodus mortoni*	Osteichthyes	Campanian	11.2 m^[^^150^^]^
	Cenozoic	*Odobenus rosmarus*	Mammalia	Recent	3.8 m, 1883 kg^[^^8^^]^
Chemosymbiotic marine shell-bearing animals	Mesozoic	*Capsiconcha withami*	Bivalvia	Barremian	300 mm^[^^151^^]^
	Cenozoic	*Bathymodiolus boomerang*	Bivalvia	Recent	370 mm^[^^152^^]^
Solitary photosymbiotic marine animals	Palaeozoic	*Alatoconcha sp*.	Bivalvia	Middle Permian	1 m^[^^153^^,^ ^154^^]^
	Mesozoic	*Titanosarcolites sp*.	Bivalvia	Maastrichtian	2 m^[^^155^^]^
	Cenozoic	*Tridacna gigas*	Bivalvia	Recent	137 cm^[^^156^^]^
Marine pelagic planktivores	Palaeozoic	*Titanichthys sp*.	Placodermi	Late Devonian	2.5 m^[^^157^^,^^158^^]^
	Mesozoic	*Leedsichthys sp*.	Osteichthyes	Callovian	9 m^[^^159^^]^
	Cenozoic	*Balaenoptera musculus*	Mammalia	Recent	33.5 m; 140,000 kg^[^^8^^,^ ^120^^]^
Marine benthic suspension-feeders	Palaeozoic	*Gigantoproductus sp*.	Brachiopoda	Visean	375 mm^[^^160^^]^
		*Macrotheca almgreeni*	Hyolitha	Late Permian	50 cm^[^^161^^]^
	Mesozoic	*Platyceramus platinus*	Bivalvia	Campanian	3 m^[^^162^^]^
	Cenozoic	*Pinna nobilis*	Bivalvia	Recent	571 mm^[^^163^^]^

The increase in maximum size is in line with overall trends toward greater size over time. In the Ediacaran period, the largest organisms so far known are the frond-like erect osmotrophic *Charnia*, reaching a height of 2 m above the seafloor [19, 20] and the sediment-feeding organism *Nilpenia rossi*, with a diameter of 30 cm [21]. The largest animals of the succeeding Early Cambrian period are predatory anomalocarids, which reached a length of 1 to perhaps 2 m [22]. The category of very large, mobile filter-feeders was ushered in during the early Ordovician with anomalocaridids almost 1 m long [23, 24]. The maximum height of sedentary suspension-feeding animals above the seafloor was 50 cm for Early Cambrian sponges [25], rising to 1 m or more for Late Silurian and later Palaeozoic crinoids [26]. Mean and maximum body sizes increased from the Middle to the Late Ordovician and again from the Silurian to the Devonian in brachiopods [27], and from the Late Ordovician to the Early Devonian in deep-water arthropods, echinoderms, and brachiopods [28]. Maximum size among pelagic apex predators rose steadily from their first post-Permian appearance in the Early Triassic to at least the Early Cretaceous [29]. At the largest timescales, Payne and colleagues [30] documented two stepwise increases in maximum size, the first at 1.9 Ga coinciding with the origin of the eukaryotic cell and the second beginning 0.6 Ga with complex animal multicellular organization. All available evidence indicates that decreases in maximum size during and immediately following mass extinctions were temporary and that they did not significantly slow the upward trend in maximum size of organisms in most ecological categories.

Plants likewise show a general upward trend in maximum size. During the Palaeozoic, there was an increase in known maximum tree height from 8 m in the Givetian stage (385 Ma) of the Middle Devonian [31] to 40 m in the Famennian stage (365 Ma) of the latest Devonian [32] and 48 m in upland forests of the Bolsovian stage of the Late Carboniferous [33]. Osborne and Beerling [34] suggested on the basis of physiological models that high levels of CO_2_ in the atmosphere should have enabled very tall trees to grow throughout much of the world during the Cretaceous, especially in the tropics and temperate coastal regions. The tallest living trees (the southeast Australian *Eucalyptus regnans*, 114.5 m; and the Californian redwood *Sequoia sempervirens*, 115.6 m) [35] reach greater heights than any known fossil species, although the record is far from perfect. Extraordinarily long vines, such as the 240 m long palm cited by P. W. Richards [36] from an Indonesian rain forest, indicate the potential for land plants other than trees to achieve great size. The largest marine plants (the brown laminarialean kelps *Macrocystis pyrifera* and *Nereocystis luetkeana* from the northeastern Pacific, reaching lengths of 45.6 and 40 m respectively) [37] are also geologically young. Molecular phylogenetic studies show that these species are derived members of their clade, and that they are no older than Middle Miocene [38, 39]. In fact, brown and red algae with large foliose thalli date back only to the Early Cretaceous [38].

The size increases that ultimately led to era-level gigantism were neither monotonic nor uniform. Large-bodied species suffered selective extinction during all known major crises [1, 40–43] and were conspicuously absent on land for 8 to 10 m.y. after the end-Permian extinction [1, 44]. Extremely rapid size increases after the unstable Early Triassic, however, resulted in Middle Triassic herbivorous and predatory vertebrates that exceeded their Permian counterparts on land in size [1, 17, 45]. Giant marine ichthyosaurs rivaling the size of Late Paleozoic open-water predators had evolved 3 to 4 m.y. after the first appearance at 284 Ma of marine reptiles [29, 46, 47].

Such rapid attainment of large size notwithstanding, extreme gigantism at the era or Phanerozoic scale required a much longer time to evolve [17]. My analysis of the ages of era-level giants in Table 1 indicates that the interval from the last mass extinction to the age of the largest era-level species is approximately 77 +/- 22 m.y. for the Paleozoic (8 categories), 98 +/- 40 m.y. for the Mesozoic (10 categories), and 59 +/- 11 m.y. for the Cenozoic (10 categories). It should be noted, however, that the intervals are not normally distributed. Considering only Phanerozoic-level giants, these animals appeared 124 +/- 17 m.y. after the last mass extinction for the Mesozoic (4 categories) and 63 +/- 2 m.y. for the Cenozoic (6 categories).

Traits that have been hypothesized to enable gigantism existed long before maximum size was realized. Long necks, which perhaps predisposed sauropod dinosaurs to become gigantic [16], already characterized the clade Sauropodomorpha during the Late Triassic, at least 100 m.y. before the mid-Cretaceous global giants in this clade of herbivores existed. The zooplankton-straining baleen, which is associated with gigantism in living balaenopterid mysticete whales, evolved some 30 Ma during the Late Oligocene, long before mysticetes became very large in the Pleistocene [48–50]. Chemosymbiotic mytilids originated as small (less than 50 mm long) bivalves during the Late Eocene, 35 m.y. before they achieved the enormous sizes of some living species [51–53]. The photosymbiotic bivalve *Tridacna gigas* appeared in the Late Miocene, 15 to 20 m.y. after the genus *Tridacna* first appears in the fossil record in the Late Oligocene [54]. One-way respiratory ventilation, which perhaps permitted gigantism in dinosaurs and pterosaurs [16, 55], characterizes diapsid reptiles generally [56], and therefore preceded global gigantism in these diapsid clades by as much as 200 to 230 m.y.

Taken together, these data imply that the circumstances leading from large size to out-sized gigantism are either highly unusual or exceptionally long lasting. Either way, enabling factors and selection for large size must coincide and mutually reinforce each other.

### Trophic Levels, Land and Sea

Burness and colleagues [57] showed that the largest herbivorous terrestrial vertebrates are 5 to 33 times more massive than their predatory counterparts, the factor of difference depending on whether comparisons are for endotherms or ectotherms. This maximum-size advantage of herbivores has held throughout most of the history of terrestrial ecosystems. Exceptions are the intervals from the Silurian to the earliest Permian and from the Anisian to the Norian epochs of the Triassic [58, 59], when predators exceeded herbivores in maximum size. The Paleocene epoch may also be an exception if the gigantic snake *Titanoboa cerrejonensis* (length 13 m, estimated mass 135 kg) [60] was at least partly a terrestrial predator.

The largest marine animals from the Early Cambrian onward have been pelagic, either apex predators or zooplanktivores (Tables 1 and 2). Herbivores have been the largest bottom-dwelling marine animals since at least the Late Oligocene, with the evolution of desmostylians and the Early Pliocene replacement by large sirenians [61, 62], and perhaps since the Late Cretaceous if some sea turtles were herbivorous at that time [63].

**Table 2:** Largest Phanerozoic members of major clades not already listed in Table 1.

Category		Clade	Age	Size
Terrestrial	*Birgus latro*	Crustacea	Recent	300 mm^[^^164^^]^
	*Pebasiconcha immanis*	Gastropoda	Middle Miocene	256 mm^[^^165^^]^
	*Eryops macrocephalus*	Amphibia (aquatic)	Early Permian	2 m^[^^166^^]^
	*Megalania prisca*	Squamata	Pleistocene	2000 kg^[^^167^^]^
	*Geochelone sp*.	Chelonia	Recent	1000 kg^[^^97^^]^
	*Argentavis magnificens*	Aves (flying)	Late Miocene	70 kg^[^^168^^]^
	*Aepyornis maximus*	Aves (non-flying)	Holocene	275 kg^[^^169^^]^
Marine	*Isotelus rex*	Trilobita	Late Ordovician	700 mm^[^^164^^]^
	*Jaekelopterus rhenaniae*	Chelicerata (and Arthropoda)	Late Oligocene	2.5 m^[^^170^^,^^171^^]^
	*Jasus edwardsii*	Crustacea	Recent	60 cm^[^^172^^]^
	*Odontodactylus scyllarus*	Crustacea	Recent	60 cm^[^^164^^]^
	*Campanile parisiensis*	Gastropoda	Middle Eocene	1 m^[^^8^^]^
	*Cryptochiton stelleri*	Polyplacophora	Recent	350 mm^[^^3^^]^
	*Dunkleosteus terrelli*	Placodermi	Late Devonian	7 m^[^^173^^]^
	*Carcharocles megalodon*	Chondrichthyes	Middle Miocene-Late Pliocene	18m^[^^135^^]^
	*Rhincodon typus*	Chondrichthyes	Recent	18 m^[^^174^^]^
	*Archelon sp*.	Chelonia	Maastrichtian	4 m^[^^175^^]^

It is striking that, at the level of global Phanerozoic gigantism, maximum size in the three terrestrial categories was achieved in extinct taxa, whereas that in 6 of the 7 marine categories was not reached until the Late Neogene (Table 1). Although the numbers are too small to permit an evaluation of statistical significance, the difference between marine and terrestrial global gigantism is all the more surprising in view of the much longer history of marine multicellular animals.

Phylogeny.—Before considering whether extreme giants exhibit general properties and whether particular circumstances favor their evolution, it is important to ascertain to what extent historical contingency (the effects of initial conditions, including membership in particular clades) explains the distribution and characteristics of era-level giants. Of the 20 era-level giants (predators, herbivores and open-water planktivores) considered in Table 1, 17 (85%) are vertebrates, 2 (10%) are molluscs and 1 (5%) is an arthropod. For the Mesozoic and Cenozoic eras, the dominance of vertebrates is 93% (13 of 14 species). The single clade Diapsida accounts for 4 Mesozoic and 1 Cenozoic era-level giants, whereas the clade Therapsida + Mammalia accounts for 8 species (2 in the Paleozoic, 6 in the Cenozoic). Table 1 records 13 cases of succession from one era-level giant to another within the same ecological category of mobile animals, albeit with a long time gap between pairs. All 13 represent shifts among major clades.

For sedentary marine era-level giants (chemosymbiotic, photosymbiotic and benthic suspension-feeding shell-bearing animals), 8 of 9 (89%) are molluscs (7 bivalves and one hyolith) and 1 is a brachiopod. The 5 cases of successive era-level giants in the same ecological category of sedentary animals all represent switches from one major clade (including among bivalve clades) to another.

These data indicate that, although a few class-level clades dominate the ranks of era-level giants (diapsids in th~ Mesozoic, mammals in the Cenozoic, and bivalves in both eras), there is no evidence that extreme gigantism is retained within a clade in successive eras. Paleozoic marine brachiopods and cephalopods and flying insects became era-level giants, as did Mesozoic ground-dwelling and marine diapsids, but they have not done so during the Cenozoic despite persisting as class-level clades. Clade membership is important not because a clade has properties conducive to the evolution of large size, but because individual species acquire traits and live under circumstances that are compatible with gigantism.

Productivity.—In order to maintain a very large body, an animal must have access to sufficient food within its reach. There should therefore be a general correspondence between maximum body size and the productivity of the environment, the rate at which accessible food becomes available. For any given level of productivity, maximum body size will increase according to the area or volume of habitat that is sampled for food, as long as the energy expended does not exceed the potential energy gained. Very large animals with high food requirements should therefore be highly mobile or, if sedentary, be able to pump large amounts of water in order to avoid local depletion [64].

Earlier work indicated that regional-scale primary productivity in the coastal benthos is positively correlated with maximum body size in herbivorous molluscs, and that nearshore planktic productivity correlates with maximum size in suspension-feeding bivalves and gastropods [3, 65, 66]. Today's largest marine herbivores (sirenians) live(d) in highly productive kelp forests and seagrass meadows where the standing stock of primary producers is high and accessible. Coral reefs are also productive but have lower standing stocks of photosynthesizers, which are usually toxic photosymbioses or calcareous algae that are not readily consumed. Herbivores on reefs are notably small compared to those in kelp and seagrass beds. In the open ocean, planktivores and apex predators integrate productivity on a basin-wide or even global scale [67, 68].

Although regional and global marine primary productivity in the past cannot be estimated directly, evidence from many sources indicates a general rise through the Phanerozoic, with large increases from the Cambrian to the Ordovician, during the Devonian, in the later Mesozoic, and the Late Neogene [3, 23, 66, 69]. The Late Mesozoic (Late Jurassic to Late Cretaceous) could represent a peak in planktic productivity as indicated by the evolution of very large deposit-feeding nerineoidean gastropods which reached lengths of 50 cm beginning in the Late Jurassic [70], and by a size maximum in suspension-feeding bivalves in the Late Cretaceous (Table 1) and of suspension-feeding gastropods (*Gigantocapulus schmitti*, length 400 mm) [71, 72]. Globally high planktonic productivity coincided with times of widespread uplift, volcanism and attendant high rates of chemical weathering and sediment input from the land [73–75]. These inputs of nutrients were magnified by increasing rates and intensities of bioturbation and other forms of nutrient cycling (73–75]. In short, global and era-level gigantism in organisms relying on a planktonic food supply seems to coincide with peaks in planktic productivity as indicated by independent evidence, although precise correspondences are still beyond the resolution of currently available data.

Primary production on the seafloor might have remained low until the evolution of seagrasses in the Late Cretaceous (Campanian stage) and of large temperate-zone brown algae in the Late Paleogene. Certainly no large animal that can be inferred to have been herbivorous occurred in the sea before the Late Jurassic (Table 2).

A special class of primary producers/consumers comprises marine animals containing microbes capable of fixing carbon from inorganic sources. Such symbiotic associations have frequently led to gigantism [76–80]. Most shell-bearing chemosymbiotic animals, which house methanotrophic and/or sulfide-oxidizing bacteria, have greatly reduced or eliminated their reliance on suspended food in favor of feeding on their symbionts, but chemosymbiotic bathymodioline mytilid mussels and all photosymbiotic bivalves augment suspension-feeding or particle-feeding with nutrition from their symbionts [79]. Known or suspected photosymbiotic bivalves were the largest bottom-dwelling suspension-feeders of their time during the Silurian, Permian, Triassic, Jurassic, latest Cretaceous (Maastrichtian) and Late Miocene to Recent [80]. This may also have been the case in the Late Eocene if the poorly known *Somalidacna lamellosa* from Somalia (length 450 mm) [81] was photosymbiotic, because this species reached a size similar to that of the more or less contemporaneous Antarctic nonsymbiotic *Perna* sp. [82, 83]. Chemosymbiotic shell-bearing animals rarely reached sizes as great as those of the largest contemporary suspension-feeders or photosymbiotic counterparts, but they are still very large. Because of their unique trophic status, I have treated these symbiotic animals in categories separate from other ecological groups. As many authors have emphasized, symbiotic associations can also occur in very small-bodied animals [76, 77].

The correspondence between maximum size of herbivores and primary production and size of land plants remains to be documented in detail but appears to be weak or negative. Evidence from leaf-vein densities and properties of the vascular system indicates that mid-Cretaceous to Recent angiosperms (especially eudicots and grasses) became more productive than other fossil and living land plants [84]; yet the largest herbivores (sauropods) existed at times when these productive plants had not yet achieved ecological dominance. Indeed, the consistently low protein content of Mesozoic vegetation [85] enabled or even propelled lineages of large-bodied dinosaurs to become gigantic as a means of acquiring enough nutrients from a low-quality food [85]. The largest Cenozoic herbivores are mammals in grasslands and savannas, which can be productive but which fall short of the productive capacities of some forests. Maximum sizes and metabolic rates of forest-dwelling herbivorous mammals are lower than those in more open environments [86]. Large ground-dwelling herbivores cannot easily maneuver among large, closely spaced trees, and are unable to reach canopy leaves and branches. Herbivores that live in or fly among trees are limited in maximum size in comparison to their ground-dwelling counterparts.

Climate.—Opposing arguments make contrasting predictions about the climates under which gigantism evolves. High oxygen solubility and low oxygen demand in cold water should make polar regions and the deep sea (and deep cold lakes) ideal for the establishment of gentle giants [87]. The huge Early Ordovician anomalocarids and trilobites from Morocco and Portugal, which at that time were situated at high southern latitudes, were explained by the low temperatures in which they lived [23, 88]. On the other hand, although the demand for oxygen increases at higher temperatures, so do diffusivity of oxygen and general activity levels [89]. In addition, the largest animals in all trophic and habitat categories maintain a high body temperature either by producing copious body heat or by living in a warm place.

On the whole, the evidence indicates that climate plays an indecisive role as an enabling factor for extreme gigantism. An analysis of the climatic distribution of the Cenozoic-level giants listed in Table 1 shows that 3 are tropical (including *Bathymodiolus boomerang*, associated with hydrothermal vents), 2 are from cold regions and 2 are widespread. Two of these species are the largest global members of their respective clades. When these two species are added to the list of largest living members of the clades considered in Tables 2 and 3, I considered a total of 35 species belong to 32 clades (with 3 clades each represented by 2 largest species). Of the 35 species, 22 (63%) are tropical, 10 (29%) are from cold climates, and 3 (8.6%) are climatically widespread. This distribution pattern is similar to the pattern for all species regardless of size.

**Table 3:** Largest living species in major clades not listed in Tables 1 and 2.

Category	Clade	Taxon	Habitat	Size
Marine				
	Brachyura	*Pseudocarcinus gigas*	temperate	400 mm, 14 kg ^[^^176^^]^
	Isopoda	*Bathynomus giganteus*	deep sea	50 cm^[^^8^^]^
	Paguroidea	*Tisea grandis*	tropical	128 mm^[^^177^^]^
	Cirripedia	*Austromegabalanus psittacus*	temperate	300 mm ^[^^178^^]^
	Amphipoda	*Megaceradocus gigas*	deep sea	570 mm ^[^^164^^]^
	Xiphosurida	*Tachypleus gigas*	tropical	450 mm ^[^^164^^]^
	Brachiopoda	*Magellania venosa*	temperate	85 mm ^[^^178^^]^
	Gastropoda	*Syrinx aruanus*	tropical	722 mm^[^^8^^]^
	Scaphopoda	*Fissidentalium metivieri*	tropical	180 mm ^[^^179^^]^
	Cephalopoda	*Architeuthis dux*	deep sea	19 m ^[^^10^^]^
	Cephalopoda	*Mesonychoteuthis hamiltoni*	polar	200 kg ^[^^13^^]^
	Asteroidea	*Pycnopodia helianthoides*	temperate	1.3 m^[^^3^^]^
Terrestrial				
	Brachyura	*Cardisoma carnifex*	tropical	150 mm ^[^^164^^]^
	Scorpionida	*Hadogenes troglodytes*	tropical	21 cm^[^^180^^]^
	Araneae	*Heteropoda maxima*	tropical	46 mm, 300 mm (legspan)^[^^181^^]^
		*Theraphosia blondi*	tropical	11.9 cm, 28 cm (legspan) ^[^^182^^]^
	Diplopoda	*Archispirostreptus gigas*	tropical	82 g ^[^^183^^]^
	Chilopoda	*Scolopendra gigantea*	tropical	241 mm^[^^184^^]^
	Arthropoda	*Phobaeticus chanii*	tropical	357 mm^[^^185^^]^
	Orthoptera	*Deinacrida heteracantha*	temperate	82 mm^[^^186^^]^
	Coleoptera	*Titanus giganteus*	tropical	17 cm ^[^^14^^]^
	Lepidoptera	*Ornithoptera alexandraea*	tropical	25 cm ^[^^187^^,^ ^188^^]^ (wingspan)
	Odonta	*Petalura ingentissima*	tropical	170 mm wingspan, 125 mm length ^[^^189^^]^
	Gastropoda	*Archachatina marginata*	tropical	213 mm ^[^^169^^]^
	Amphibia	*Andrias davidianus*	temperate	160 cm, 50 kg ^[^^190^^]^
	Archosauria	*Crocodylus intermedius*	tropical	6.25 m^191^
	Serpentes	*Python natalensis*	tropical	9 m ^[^^192^^]^
	Mammalia	*Loxodonta africana*	tropical	8000 kg ^[^^193^^]^

This conclusion is supported by analyses of gigantism in fossil and living mammals, although the authors of these studies expressed their results differently. According to these authors, maximum size in terrestrial mammalian lineages are concentrated in time during the warm Middle Eocene, the cooler Oligocene and the variably warm and cool Pliocene to Recent interval [7, 90, 91]. A problem with these and many other studies is that differences in mean global temperature, such as between the Eocene and Pleistocene, obscure the fact that tropical conditions have always existed during the Phanerozoic. Global cooling with the advent of widespread glaciation in the Pleistocene affected the tropics, but many living giants such as surviving elephants, rhinoceroses, snakes, turtles, lizards, crocodiles, insects, spiders, crabs, hermit crabs, stomatopods, horseshoe crabs, recently extinct ground sloths, and extinct South American giant rodents are tropical in distribution.

In any case, the largest Eocene mammals are smaller than their later counterparts of the Late Oligocene and Pleistocene.

Among land plants, the tallest trees occur at temperate latitudes [92, 93], whereas the longest vines are tropical. Marine plants also reach maximum sizes along temperate shores.

Metabolism and Oxygen.—The single most important trait that affects the body size of organisms is metabolic rate. Two metabolic categories of very large animals can be distinguished: (1) the so-called gentle giants, animals with low metabolic demand, sluggish habits, slow growth and often long lifespans; and (2) active animals with high metabolic rates and energy requirements, thermal control and rapid growth. Giants of the two groups are adapted to contrasting circumstances.

Animals in the first category are local giants such as the colossal squid [13] and other Antarctic animals [94, 95] (isopods, pycnogonids and nudibranchs), deep-water crustaceans in Lake Baikal [96], and tortoises on oceanic islands [97]. These animals occupy cold or unproductive environments where threats from metabolically active species are low or intermittent.

All the largest members of trophic and habitat categories throughout the Phanerozoic belong to the second group of highly active animals. Even among sedentary bottom-dwelling suspension-feeders, it is the species with the strongest ciliary currents generated by the filtering gills that achieve the largest sizes [3]. The large terrestrial and marine reptiles of the Mesozoic were likely mesotherms, which maintained high body temperatures by virtue of their large size [1, 98–106] and whose metabolism approached that of endotherms.

At first glance, this pattern does not make sense. Heat production, high activity levels and rapid metabolism are costly and should therefore place constraints on the attainment of large body size. However, these traits make it possible for large animals to cover great distances quickly in search of valuable but widely scattered resources [2, 107]. The equivalent for current-generating suspension-feeders is to sample large volumes of water. Active ventilation relieves limits on large size in arthropods and vertebrates [108, 109].

Large active animals require abundant oxygen together with effective means of distributing it throughout the body. This truism led to the hypothesis that gigantism in Late Paleozoic arthropods on land was made possible by oxygen levels in the atmosphere that were 20% to perhaps 33% higher than today's [110–113]. Successive episodes of oxygenation of the atmosphere in the latest Neoproterozoic, Late Cambrian and Devonian [30, 114–116] coincided with the evolution of more active animals and with increases in maximum size. Note, however, that the largest Paleozoic members of major marine clades (Cephalopoda, Trilobita, Eurypterida and Placodermi) lived before the Late Paleozoic oxygen peak [117]. The post-Paleozoic history of oxygen remains controversial. Some models [118] indicate a secondary maximum in oxygen during the mid-Cretaceous, roughly coinciding with global gigantism in terrestrial herbivores; but other models [119] indicate a low point in oxygen at that time. The available qualitative evidence suggests that gigantism at the Phanerozoic scale in ecological categories is not closely correlated with oxygen levels [117]. The early evolution of efficient one-way ventilation in diapsid reptiles [56] could have made vertebrate physiology and maximum size less strictly limited by oxygen concentrations and enabled very large size to develop even at modest oxygen levels [16].

Habitat Size.—Very large mobile animals with large appetites require extensive home ranges and wide species-level geographic distributions in order to maintain viable populations. This is especially true for animals such as mammals with very long gestation times [120, 121]. Cenozoic endotherms therefore reached maximum sizes on the largest land masses [57, 90J. The mega-continents of the Mesozoic could have enabled gigantism in dinosaurs and pterosaurs [16], but it is notable that the Late Paleozoic to Early Triassic supercontinent Pangaea did not support land animals anywhere near as large as later Mesozoic or Cenozoic vertebrates. Conditions in the interior of Pangaea were likely too dry and too unproductive [122] to support very large land animals.

In the sea, very large vertebrates have significantly larger species-level geographic ranges than their smaller counterparts [123]. All Cenozoic-level giants in this category occur in more than one ocean basin.

The effect of habitat size on gigantism is not apparent in sedentary organisms such as bottom-dwelling suspension-feeders, seaweeds and land plants. These organisms can maintain higher population densities than mobile gigantic vertebrates. The tallest trees—redwoods from southern Oregon to central California and *Eucalyptus regnans* from southeastern Australia—occupy notably small geographic areas. The large brown alga *Macrocystis pyrifera* today occupies both the North Pacific and parts of the Southern Ocean, but it (and its large size) originated in the North Pacific on the American side, as did the nearly equally large *Nereocystis leutkeana*. The largest living gastropods, hermit crabs, bivalves (in 3 habitat categories) and asteroids (Tables 1–3) are also geographically limited to small ranges.

In short, although the extent of suitable habitat correlates with maximum sizes in vertebrates that are already large and that have high metabolic rates, the causal link between gigantism and geographic range is indirect. The species-wide property of geographic range is at best an inconsistent indicator of, and enabler for, the attainment of local or global gigantism.

Predation and Competition.—The factors considered above—productivity, climate, oxygen and habitat size—belong to the category of enabling factors [74J, agencies that permit but do not compel very large body size to evolve. Agencies that select in favor of large size propel some lineages toward gigantism to the extent that the enabling factors allow [74]. Given that large size often confers advantages in competition and in defense against lethal predation, it is reasonable to propose that predators and competitors are the primary agents selecting for large size in some lineages. Extreme gigantism would then imply that such selection is either extremely intense or highly consistent for very long periods of time. Note that most lineages will not be subject to such intense or enduring selection either because their representatives fail as competitive dominants or are severely limited by trade-offs with other important functions [74]. Incidentally, this same argument casts doubt on the generality of Cope's Rule (see Methods).

Predation (the consumption of part or all of an organism by an animal) is a universal ecological interaction and selective agency. Many victim species grow to a refuge in large size from predation, indicating the antipredatory benefit of large size. The observation that the largest terrestrial herbivores reach greater maximum sizes than their predatory counterparts at most times (see above) is consistent with such selection. The 5-fold or greater disparity between the maximum size of herbivores and predators, however, indicates that predation is unlikely to be the only, or even the most important, agency favoring extreme gigantism. In the unusual case of human superpredators, no species exploited by us in the wild can reach a size refuge, so that large size has become a liability for victim species everywhere [124–128].

Competition in the strict sense (individuals or groups attempting to acquire or defend resources without consuming each other) is also universal. Dominance in competition for food, shelters or mates in animals and for light and water in trees is often associated with large size [2, 129–132] and, unlike predation, does not diminish even at very large size. Predation may therefore have been important early in the evolution of size increase in lineages that ultimately became gigantic at the era or Phanerozoic level, but competition likely pushed these lineages to their maximum size. I speculate that competition for mates will have been especially important for large terrestrial and marine vertebrates.

## General Discussion

Having considered the factors that either enable or compel large body sizes to evolve, I now ask how these factors can explain the observed historical pattern in era-level and Phanerozoic-level global gigantism. In particular, (a) what distinguishes the post-Paleozoic interval (especially the second halves of the Mesozoic and Cenozoic eras), during which all ecological categories witnessed the evolution of their largest representatives, from the Paleozoic? and (b) what accounts for the retreat from global gigantism of terrestrial animals during the Cenozoic while maximum size in most marine categories reached its peak during the Late Cenozoic?

Part of the answer to the first question resides in differences in the rate, access to, and fate of primary production for which consumers compete. During the Late Paleozoic, tropical conditions and abundant oxygen would seem ideal for the evolution of gigantism; but evidence from leaf venation indicates that terrestrial productivity was low [84] even though at least some lycophytes (tree club mosses) had carbon-concentrating mechanisms [133]. Terrestrially generated production was buried rather than rapidly decomposed or consumed by large herbivores before the Early Permian, and much of it was in the form of indigestible organic compounds [134]. The interdependencies among producers, herbivores and decomposers that made nutrients accessible within terrestrial systems in later eras were not yet in place before the Permian [74]. These interdependencies began to develop during the Permian, but were temporarily disrupted by the end-Permian catastrophe. Their reappearance and greater development by Middle Triassic time provided conditions for more productive plants and larger consumers to evolve. These terrestrial shifts would have affected marine ecosystems as well, especially when worldwide tectonic activity and the colonization of marine soft-bottom environments by Late Cretaceous angiosperms enhanced nutrient supply and cycling [74].

Global size maxima in terrestrial ground-dwelling herbivores and predators and flying animals (all diapsid reptiles) in the later Mesozoic have been attributed to efficient one-way ventilation in the respiratory system [16, 55], a low protein content of forage for herbivores [85], and in the case of herbivorous sauropods to a very long neck, which enables these giants to reach food over a large area and height range without moving the entire body [16]. The long neck, in turn, was made possible by the habit of most diapsids of swallowing large chunks of food whole {16]. Mammals employ the less efficient in-and-out breathing with the use of a diaphragm, and chew food in the mouth, for which strong neck and masticatory muscles are necessary, limiting the length of the neck [16]. Both of these mammalian traits place more stringent limits on maximum size than in diapsids.

These constraints might collectively explain the smaller maximum sizes of Late Paleozoic and Cenozoic terrestrial animals, but two lines of argument cast doubt on the scope of this explanation. First, some marine mammals are exceptionally large despite their inefficient breathing and short necks. Second, neither the respiratory nor the chewing constraints apply to birds; yet no Cretaceous or Cenozoic bird comes close to the great size of Late Cretaceous flying pterosaurs. Important as traits such as a long neck, one-way ventilation and specialization to a low-protein plant diet might be for enabling very large size to evolve, they do not suffice to explain the smaller maximum sizes of Cenozoic as compared to Mesozoic giants on land, especially given that higher primary productivity characterized Cenozoic ecosystems. They also do not account for the observed distribution and characteristics of marine global giants.

An intriguing possibility is that gigantism as a means of achieving competitive superiority has lost its luster relative to alternative pathways of becoming a top consumer. One such alternative is social organization, and in particular cooperative hunting, in which individuals form cohesive groups that are highly effective in collective resource acquisition and defense. In particular, group hunting by relatively small predators makes even very large prey vulnerable. This form of collective predation has become relatively widespread among carnivoran mammals during the Late Cenozoic. The use of weapons has enabled our own highly social species to bring down animals of any size. Individual gigantism has in effect been replaced on land by gigantism at the group level. Large individual size will still be advantageous in competition among adults, but animals the size of Mesozoic giant sauropods, hadrosaurs and ceratopsians might be vulnerable targets for more agile Cenozoic endothermic predatory mammals, especially for cooperatively hunting ones.

Even in the sea, where social organization remains much less common than on land, there is evidence of replacement over time of a gigantic solitary apex predator (the Middle Miocene to Late Pliocene shark *Carcharocles megalodon*, length 18 m) by the much smaller (7 m) Pleistocene to Recent cooperatively hunting killer whale *Orcinus orca* [135, 136]. The much later appearance of Phanerozoic level giants in the sea than on land can therefore perhaps be ascribed to the absence of sociality not only in bottom-dwelling marine suspension-feeders, photosymbiotic and chemosymbiotic animals, but also in most large vertebrates. The reasons for this difference in group competition and defense between marine and terrestrial ecosystems remain obscure.

A second possible explanation for the spatial and temporal distribution of global giants resides in the three-dimensional structure of habitats. For mobile animals, global gigantism is achievable only in productive uncluttered environments such as the open ocean, savanna or grassland vegetation, the air space above the forest canopy, or surface waters above subtidal kelp and seagrass beds. Three-dimensionally complex habitats such as reefs or the forest understory require alternatives to very large size, or at least gigantism on a much smaller scale such as that of ground-dwelling Carboniferous arthropods. The increased cover of closed forests after the Cretaceous [137] can go some way toward explaining the absence of Phanerozoic-level ground-dwelling giants during the Cenozoic. In this connection it is interesting that Steller's sea cow (*Hydrodamalis gigas*), the largest known marine herbivore, apparently fed largely in the canopy of North Pacific kelp forests [138].

## Conclusions

The history of life is a complex tale of changing circumstances that are influenced by, and that influence, patterns of natural selection as well as the enabling factors that determine how far natural selection can go in any particular direction. In the case of global gigantism, both the tectonically controlled inputs of essential resources and the evolved ecological interdependencies that regulate how these resources are recycled through the biosphere explain why the largest animals in each of 10 trophic and habitat categories evolved after the Paleozoic era. Global gigantism as a means of achieving competitive superiority requires not only intense and sustained selection for large size, but also an ecological infrastructure that provides enough oxygen, food and stability of supply to make this possible. How such gigantism evolves and in which clades depends on physiological and anatomical innovations that are useful first in small-bodied animals in defense and feeding. Effective alternatives to extremely large size, particularly including coordinated food-gathering, became common on land but remain relatively scarce in most marine ecosystems. This difference between land and sea is also manifested by the much tighter link between primary productivity and gigantism in marine ecosystems than in terrestrial ones.

Although this paper is concerned with one trait—very large size of organisms—it illustrates the importance of evaluating fossil and living organisms in their ecological contexts and roles for understanding large historical trends. Organisms and their traits are products of interactions, which change over time as external triggers and internal regulatory mechanisms change. They are not abstractions, but real things and real properties that function in the evolving biosphere.
